# Preventing Damage Limitation: Targeting DNA-PKcs and DNA Double-Strand Break Repair Pathways for Ovarian Cancer Therapy

**DOI:** 10.3389/fonc.2015.00240

**Published:** 2015-10-26

**Authors:** Daniela A. Dungl, Elaina N. Maginn, Euan A. Stronach

**Affiliations:** ^1^Molecular Therapy Laboratory, Department of Surgery and Cancer, Ovarian Cancer Action Research Centre, Imperial College London, London, UK

**Keywords:** DNA-PKcs, platinum resistance, ovarian cancer, DNA repair, chemosensitization

## Abstract

Platinum-based chemotherapy is the cornerstone of ovarian cancer treatment, and its efficacy is dependent on the generation of DNA damage, with subsequent induction of apoptosis. Inappropriate or aberrant activation of the DNA damage response network is associated with resistance to platinum, and defects in DNA repair pathways play critical roles in determining patient response to chemotherapy. In ovarian cancer, tumor cell defects in homologous recombination – a repair pathway activated in response to double-strand DNA breaks (DSB) – are most commonly associated with platinum-sensitive disease. However, despite initial sensitivity, the emergence of resistance is frequent. Here, we review strategies for directly interfering with DNA repair pathways, with particular focus on direct inhibition of non-homologous end joining (NHEJ), another DSB repair pathway. DNA-dependent protein kinase catalytic subunit (DNA-PKcs) is a core component of NHEJ and it has shown considerable promise as a chemosensitization target in numerous cancer types, including ovarian cancer where it functions to promote platinum-induced survival signaling, via AKT activation. The development of pharmacological inhibitors of DNA-PKcs is on-going, and clinic-ready agents offer real hope to patients with chemoresistant disease.

## Ovarian Cancer and Chemoresistance

Ovarian cancer is the seventh most common cancer amongst women worldwide with an incidence of 6.1 [age-standardized rate (ASR)] per 100,000, and a mortality rate of 3.7 (ASR) (1). It is the fifth highest cause of cancer-related deaths among women, accounting for more deaths than any other cancer of the female reproductive system. Ovarian cancers are classified into a number of subtypes: serous, mucinous, endometrioid, clear cell, transitional, squamous, mixed, and undifferentiated subtypes (2). Further classification is by histopathological grade, with grade (borderline, grades 1–3) associated with how quickly the tumor is likely to grow. Well differentiated or low-grade (type I) tumors are typically indolent, slow growing tumors that are often detected at early stages, and include low-grade serous, low-grade endometrioid, clear cell, and mucinous carcinomas. Poorly differentiated or high-grade (type II) disease includes high-grade serous, high-grade endometrioid, mixed mesodermal (carcinosarcoma), and undifferentiated carcinomas that tend to grow and spread more quickly (3). The majority of patients (~70%) with ovarian cancer are diagnosed with late stage high-grade serous epithelial ovarian cancer (HGSOC), with dissemination of primary tumor throughout the peritoneal cavity in most cases, and the 5-year survival for these patients is <50% (4–6).

Standard therapy for advanced ovarian cancer usually involves surgical debulking of the tumor mass followed by chemotherapy, including a platinum-containing compound. Optimal tumor debulking is critical, as postoperative residual disease strongly influences patient outcome (7). First-line chemotherapy for ovarian cancer is typically carboplatin, or under certain circumstances cisplatin, given either alone or, more commonly, in combination with paclitaxel (8). Response rates to first-line therapy are favorable; however, the relapse rate is high. The platinum-free interval (PFI: i.e., interval between end of chemotherapy and relapse) is a good indicator of response to subsequent treatment with platinum: >12 month PFI predicts favorable response to retreatment; 6–12 month PFI is regarded as “intermediate”; <6 month PFI is defined as platinum resistant with commensurate poor response rate to retreatment with platinum (9, 10). Other chemotherapeutic options, typically used following platinum-resistant relapse, include topotecan (topoisomerase inhibitor), liposomal doxorubicin (inhibitor of DNA replication), gemcitabine (replaces cytidine during DNA replication leading to tumor growth arrest), and etoposide (forms ternary complexes with DNA and topoisomerase II causing DNA strand breaks), however response rates to such alternatives remain dismal. Accordingly, elucidation of mechanisms underpinning platinum resistance is an urgent priority and may allow the development of precision strategies to reverse resistance.

The biochemical mechanisms of cytotoxicity of cis- and carboplatin involve their binding to DNA and non-DNA targets and induction of cell death through apoptosis, necrosis, or both, within the heterogeneous population of tumor cells (11, 12). Direct binding to genomic DNA (gDNA) can result in a number of lesions: the initial lesion formed is bulky platinum-DNA adducts that can mediate intra- and inter-strand crosslinks. If these are not removed but are encountered by the cells’ transcription or replication machinery, stalling of these processes can lead to the generation of DNA breaks, either single-strand DNA breaks (SSB) or double-strand DNA breaks (DSB). In response to such DNA damage, a cell can either initiate repair, or if the damage is too severe, cell cycle arrest, and/or apoptosis are initiated. This process is required for a successful chemotherapeutic response. Non-DNA targets of cis- and carboplatin include components of the cell membrane lipid bilayer, such as phospholipids and phosphatidylserine, and cytoplasmic targets such as cytoskeletal microfilaments, thiol-containing peptides, proteins, and RNA (11, 13). Furthermore, these compounds can alter the activity of enzymes, receptors, and other proteins through coordination to sulfur atoms of cysteine and/or methionine residues and to nitrogen atoms of histidine residues (14). However, the formation of adducts on gDNA are thought to be the primary lesion underlying the cytotoxic effect of these drugs.

**Figure 1:**
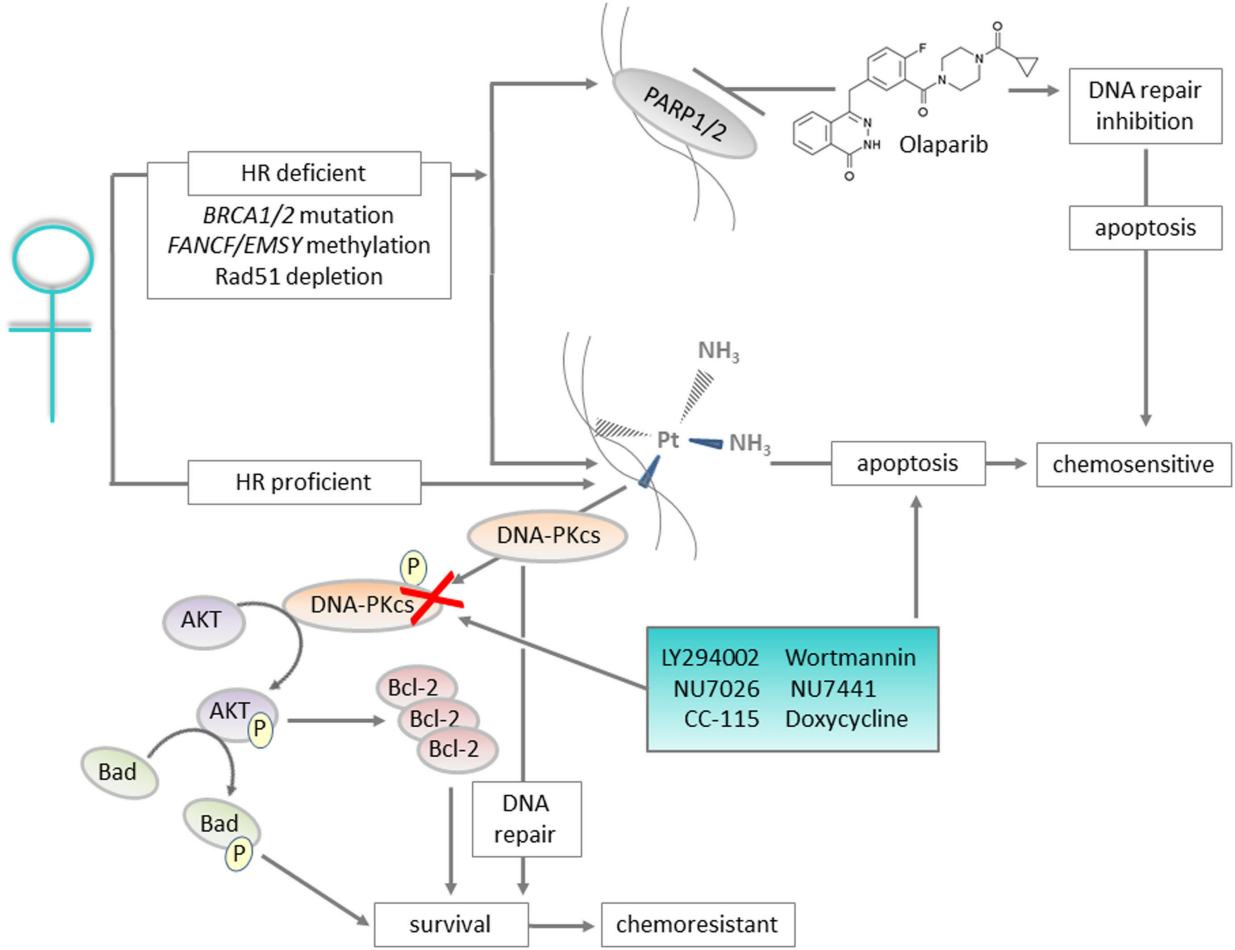
**Graphical summary: DNA repair proteins such as DNA-PKcs can be targeted to improve outcomes for patients with ovarian cancer**. Ovarian cancers with defects in the homologous recombination (HR) pathway are initially sensitive to platinum treatment, and also respond to Olaparib that targets the base excision repair pathway protein PARP. For the majority of cases, which are HR proficient, platinum-based chemotherapy is still utilized but resistance is likely. For patients with platinum-resistant disease, inhibition of DNA-PKcs, a key component of the non-homologous end joining pathway, represents a targeted approach to prevent the pro-survival AKT and anti-apoptotic signaling associated with resistance.

Given the range of targets of cis- and carboplatin, it is not surprising that resistance of ovarian cancer to these compounds is multifactorial and has been reported to involve increased drug inactivation and efflux, decreased drug influx, increased cellular glutathione and metallothionein levels, alterations in cell cycle control, oncogene expression, changes in apoptotic threshold, and increased or inappropriate activation of DNA repair pathways (9, 15, 16). Although only 5–10% of covalently bound cell-associated cisplatin is found in the gDNA, targeting the DNA repair process has shown considerable promise as a therapeutic strategy for platinum-resistant ovarian cancer. Here, we review the DNA repair network as a strategic and rational target for therapeutic intervention in ovarian cancer. In particular, we highlight the non-homologous end joining (NHEJ) pathway as an under-explored target for therapeutic discovery and use DNA-dependent protein kinase catalytic subunit (DNA-PKcs) as an example of how this pathway can be targeted therapeutically (see Figure 1 for graphical summary and Table 1 for a summary of the key messages).

**Table 1:** **Section summary and key “take home” messages**.

	Section	Key messages
1.	Ovarian cancer and chemoresistance	Ovarian cancer is the most lethal gynecological malignancy. Resistance to platinum-based chemotherapy is a major obstacle to treating patients with ovarian cancer.
2.	DNA repair proteins as therapeutic targets	DNA damage repair proteins are rational but understudied targets for developing strategies to overcome platinum resistance.
3.	Homologous recombination repair deficiency is associated with chemoresponse	Defects in homologous recombination are associated with high risk of developing ovarian cancer. These defects may also be used as theranostic markers of response to platinum- and PARP inhibitor-based chemotherapies.
4.	DNA-PKcs as a therapeutic target for ovarian cancer	DNA-PKcs is a core mediator of the non-homologous end joining pathway, which functions to repair DNA double-strand breaks. Post-translational modifications and protein–protein interactions regulate DNA-PKcs activity. DNA-PKcs inhibition has been widely associated with restoring radio- and chemo-sensitivity in a range of cancers. DNA-PKcs inhibitors developed to date are largely unsuitable for clinical use.
5.	DNA-PKcs inhibition as a platinum sensitization strategy	DNA-PKcs inhibition shows considerable promise as a strategy for reversing platinum resistance in ovarian cancer.

## DNA Repair Proteins as Therapeutic Targets

The generation of DNA damage is not an exclusive effect of chemotherapeutic agents, and occurs in response to numerous “natural” events such as replication errors or the production of reactive oxygen species after exposure to UV light. In order to respond to the various types of damage that can occur, cells possess an arsenal of DNA repair mechanisms, listed in Table 2. A more detailed description of these processes and their key components is reviewed elsewhere (17). Although DNA repair pathways are crucial for normal cell survival, defects in the execution and control of these mechanisms have been linked both with the development of cancers and response to chemotherapy. In particular, defects in nucleotide excision repair (NER), mismatch repair (MMR), and DSB repair pathways have been linked to platinum sensitivity and resistance (18, 19). Ovarian cancer together with pancreatic and breast cancers have been defined as the top three cancers in which DNA repair pathways are defective (20). As such, disruption of these pathways has been identified as a strategic approach to increase therapeutic responses to DNA-damaging agents (21–23).

**Table 2:** **DNA repair pathways and associated lesions**.

Repair pathway	Activated in response to
Nucleotide excision repair (NER)	Bulky- and helix-distorting adducts
Base excision repair (BER)	Non-helix-distorting base lesions and SSBs
Mismatch repair (MMR)	Incorrect insertion, deletion, and base misincorporation
Non-homologous end joining (NHEJ)	DNA DSBs
Homologous recombination (HR)	DNA DSBs

## Homologous Recombination Repair Deficiency is Associated with Chemoresponse

Homologous recombination (HR) is one of the major pathways for repair of DSBs, and requires an intact sister chromatid to use as a template to repair the damage. As such, it is restricted to the S and G2 phases of the cell cycle. HR deficiency (HRD) most commonly occurs in HGSOC, and is detected in 44% of these patients (24). In addition, HR defects have been suggested to drive HGSOC development (25). The majority of HRD is linked to germline or somatic alterations in the HR-associated genes *BRCA1* and *BRCA2* (~20% mutation; 10% methylation) (6, 24). Germline mutations in these genes represent significant risk factors for developing HGSOC: for a woman with a *BRCA1* mutation, the risk of developing epithelial ovarian cancer is 39–46%, and with a *BRCA2* mutation, 12–20% (26). HRD phenotype is also associated with sensitivity to platinum-based chemotherapy. Additionally, these patients are responsive to poly(ADP-ribose) polymerase (PARP) inhibitors, which are the most successful drugs targeting DNA repair proteins developed to date. PARP functions in the base excision repair (BER) pathway to repair SSBs, and inhibitors have been found to stabilize or regress ovarian cancer with *BRCA1*/*BRCA2* mutations (27–29). The biological basis for this is synthetic lethality due to loss of both BER and HR, resulting in simultaneous inhibition of SSB and DSB repair. The PARP inhibitor Olaparib was approved by the European Union for maintenance treatment of *BRCA* mutant, platinum-sensitive ovarian cancer in December 2014; the first-in-class approval for a PARP inhibitor. However, some patients without *BRCA1/2* mutations also respond to PARP inhibition, implying the presence of other HR defects. For example, BRCA pathway inactivation may also occur through methylation of *FANCF* and/or *EMSY* amplification (30, 31). Additionally, RAD51 depletion has been found to sensitize ovarian cancer cells to PARP inhibitor-based combination chemotherapy (32). For a thorough review of strategies to target HR processes, or to exploit inherent deficiencies in associated genes, with the aim of improving ovarian cancer response to platinum-based chemotherapy, see Wiedemeyer et al. (33).

DNA repair defects other than *BRCA* mutations are also probable influencers of platinum sensitivity in HGSOC; we and others have previously described a *BRCA2* reversion mutation, which does not equate to full cisplatin resistance in a HGSOC cell line series (34). The PEO1/4 cell line set were initially derived from a HGSOC patient during the platinum-sensitive and -resistant phases of the disease, respectively (35). This patient presented with a germline *BRCA2* truncating mutation, which was absent in the platinum-resistant PEO4 cell line. However, reversion of this mutation has been reported in PEO1 cells, by us and others (34, 36), and although this restores BRCA2 functionality, the platinum-resistant phenotype is not fully recapitulated; indeed, we have reported a 10-fold difference in cisplatin IC_50_ values between *BRCA* revertant PEO1 cells and PEO4 cells (34). Based on this, a continuum of platinum sensitivity can be proposed: from extreme (HR defective/*BRCA* mutant) to intermediate (HR competent i.e., *BRCA* revertant) to resistant (i.e., active resistance mechanisms). These latter mechanisms may be driven by components of other DNA repair pathway components, for example, DNA-PKcs that drives AKT survival signaling in HGSOC (37), and will be discussed now.

## DNA-PKcs as a Therapeutic Target for Ovarian Cancer

### DNA-PKcs Structure and Regulation

In comparison to HR that is restricted to post-DNA replication phases of the cell cycle, the NHEJ pathway can respond to DSBs throughout the cell cycle. This is due to its lack of requirement for a template DNA strand to use in repair. A key mediator of NHEJ is DNA-PKcs, a DNA-activated serine/threonine protein kinase that is abundantly expressed in almost all mammalian cells. DNA-PKcs is a member of the phosphatidylinositol-3-OH kinase (PI(3)K)-related protein (PIKK) superfamily, and is encoded on chromosome 8q11.21 by the *PRKDC* gene with a size of 187.07 kb and 86 exons. The DNA-PKcs protein consists of 4129 amino acids (~469 kDa), and contains a number of regions, including a catalytic domain and DNA-binding and Ku-binding domains (38).

DNA-PKcs functions as the catalytic subunit of the DNA-PK holoenzyme, which although best known for its role in NHEJ has other reported roles, including regulating apoptosis, maintaining telomere length, cell cycle control, and regulation of mitochondrial protein function (39–41). The holoenzyme is composed of DNA-PKcs and the Ku70/80 proteins. These are not constitutively associated; instead the holoenzyme is assembled in response to contact with DNA. The ring-like structure of the Ku70/80 dimer first binds to the DNA break, encircling the end and allowing the dimer to translocate along the duplex. DNA-PKcs is then recruited, and the complex acts as a scaffold, providing binding sites for downstream components of NHEJ. The kinase activity of DNA-PKcs is activated following its association with both the Ku70/80 dimer and a DNA terminus and autophosphorylation triggers a conformational change leading to the release of the DNA ends, making them available to other factors participating in DNA end-processing or ligation. As such, DNA-PKcs functions as a “gatekeeper” to protect DNA ends from premature processing, ligation, and degradation until the two broken ends are properly positioned (42–44). Although DNA-PKcs is most potently activated in response to DSBs, DNA-independent activation of DNA-PKcs due to its interaction with other proteins has also been reported, e.g., C1D, HSF1, and Lyn (45–47). The physiological significance of these DNA-independent activities of DNA-PKcs is less well understood.

DNA-PKcs contains multiple phosphorylation sites, and modifications to these alter the activity, conformation, and stability of the protein. Autophosphorylation events are initiated following binding of DNA-PKcs to DNA, and function reciprocally to coordinate the control of NHEJ. For example, phosphorylation at the ABCDE cluster, located between Thr2609 and Thr2647, promotes DNA end-processing, whereas phosphorylation at the PQR cluster between Ser2023 and Ser2056 prevents this by decreasing accessibility. End-processing of complex DSBs (e.g., hairpins) also requires trans-phosphorylation of DNA-PKcs by ATM, and this serves to recruit the endonuclease Artemis to the site of damage. Subsequent to this, autophosphorylation is required to remove DNA-PKcs in order for end-ligation to proceed (48). Autophosphorylation of DNA-PKcs at its extreme N terminus, on Ser56 and Ser72, may be involved in holoenzyme complex stability, while phosphorylation in the T-loop of the kinase domain at Thr3950 results in kinase inactivation without affecting complex stability. DNA-PKcs phosphorylation may also affect DSB repair pathway choice: cells deficient in DNA-PKcs show increased HR-mediated repair of DSBs, and autophosphorylation of DNA-PKcs at the Thr946 and Ser1004 cluster seems to serve as a switch between NHEJ and HR (49–51).

The choice of pathway may additionally be affected by the interaction of DNA-PKcs with other proteins. For example, the kinase and proto-oncogene c-Abl is phosphorylated by ATM in response to ionizing radiation (IR) and certain other DNA-damaging agents, and has been shown to interact with DNA-PKcs. Phosphorylation of DNA-PKcs by c-Abl inhibits its ability to form a complex with DNA, whereas phosphorylation of c-Abl by DNA-PKcs potentially activates c-Abl kinase activity in response to IR exposure. This suggests the existence of an autoregulatory negative feed-back loop that might lead to repression of DNA-PKcs activity after the appropriate DNA damage signaling and/or repair pathways have been initiated (52). The protein kinase activity of DNA-PKcs can also be stimulated by PARP independently of the Ku70/80 complex (53) suggesting that PARP, in addition to its key role in BER, may additionally facilitate DNA DSB repair via regulation of DNA-PKcs.

A number of mechanisms by which DNA-PKcs activity is attenuated have been reported. DNA-PKcs inactivation via cleavage by the ICE family cysteine proteases, for example, caspase-3, may occur in apoptotic cells to prevent repair of the fragmented gDNA that is produced during the final steps of the apoptotic pathway (54, 55). Degradation of DNA-PKcs via an ubiquitin-mediated proteasome pathway has also been reported following Herpes simplex virus type 1 infection, possibly as a mechanism for aiding virus replication (56).

### DNA-PKcs and Chemoresistance

Numerous studies have shown a correlation between DNA-PKcs expression and activity with response to radio- and chemotherapy. This is unsurprising given that DNA-PKcs plays a crucial role in the repair of the DSBs generated by these treatments. Additionally, chemoresponse has been linked to other activities of DNA-PKcs, including cell cycle control and regulation of mitochondrial heat-shock proteins (39, 41). However, predictive association of DNA-PKcs expression/activity in human cancer is controversial and differs between stage and pathological type. For example, cells with defective DNA-PKcs activity show increased radiosensitivity, and in lung carcinoma cell lines after γ-irradiation, the lowest DNA-PKcs protein content and kinase activity was found in the most radiosensitive cells, U-1285 and H-69, while the highest was found in the most radioresistant cells U-1810 (57, 58). DNA-PKcs activity in peripheral blood lymphocytes from untreated patients with advanced breast and uterine cervix cancers have been found to be significantly lower than in those with early stage disease. However, other studies have shown correlation between increased DNA-PKcs expression and advanced tumor stage. A genome-wide copy number and expression microarray analysis of gastric cancer revealed diverse chromosomal region alterations for different stages or histological subtypes of this disease: copy number gains at chromosome 8q11-q24 were very frequent (63%) and candidate genes such as *PRKDC* showed co-regulation with increased expression levels (59).

Some relationship may exist between the NHEJ pathway and cellular sensitivity or resistance to DNA damage; however, this relationship does not appear to be universal and may be cell or tumor-type specific. No strong correlation between radiosensitivity and DNA-PKcs was found in a study of sporadic human ovarian cancer cell lines. The data suggested that DNA-PKcs copy number, expression level, or kinase activities are not reliable predictors of radiosensitivity in ovarian cancer (60). However, inhibition of DNA-PKcs has been shown by us to reverse cisplatin resistance in a panel of ovarian cancer cell lines (37). This study used paired cell lines derived from patients before and after the emergence of platinum resistance. Interestingly, we found that DNA-PKcs inhibition did not increase the apoptotic effect of platinum in already sensitive cells. The mechanism underpinning the response to DNA-PKcs inhibition was shown to result from direct modulation of AKT survival signaling by DNA-PKcs. In platinum-resistant cells only, AKT was activated in response to cisplatin-mediated DNA damage by phosphorylation on serine 473 by DNA-PKcs. This was prevented by DNA-PKcs inhibition and doing so reversed the platinum-resistant phenotype, identifying a specific role for DNA-PKcs in mediating platinum resistance in HGSOC. DNA-PKcs-driven AKT signaling has also been identified to underlie doxorubicin resistance in glioblastoma cell lines (61).

### Pharmacological DNA-PKcs Inhibitors

Despite the current lack of a robust predictive biomarker of response to DNA-PKcs inhibition, many small-molecule inhibitors for this protein have been developed. The chemistry of these compounds varies, and some of these have selectivity for DNA-PKcs while others are broad-range PI3K family member inhibitors. The most common PIKK proteins targeted by these compounds are mTOR, PI3K, ATM, and ATR. The first identified inhibitors of DNA-PKcs were the PI3K inhibitors wortmannin and the quercetin derivative LY294002. Wortmannin is a fungal metabolite, and was found to inhibit DNA-PKcs in a non-ATP-competitive manner, to potentiate IR-induced cytotoxicity and to inhibit DSB repair at concentrations that inhibit DNA-PKcs activity. However, the IC50 required for this is ~2-fold higher than that required for PI3K inhibition, indicating the effects observed may be due to multiple protein targeting (62). The cell-permeable compound LY294002, on the other hand, acts on the ATP-binding site of PI3K proteins and inhibits DNA-PKcs in a competitive manner. The apoptotic rate of X-ray irradiated HeLa cells after pre-treatment with LY294002 was found to be significantly higher than that of untreated cells, with a prolonged G2/M delay also observed in these cells (63). IC87361 is a morpholino-flavonoid that has been derived from LY294002, and which is 50-fold more selective for DNA-PKcs than for PI3K and other kinases. It enhances radiation sensitivity in wild-type C57BL6 mouse pulmonary endothelial cells but not in SCID mouse cells, which lack DNA-PKcs. Furthermore, it was found to increase irradiation-induced apoptosis in lung cancer and melanoma cells, and caused significant growth delay in Lewis lung carcinoma xenografts treated with radiation and IC87361 in comparison to tumors treated with radiation alone (64). Other compounds developed and that have been studied as radiosensitizers are PI-103 (65), IC86621 (66), and AMA37 (67).

Two of the most widely studied DNA-PKcs inhibitors are NU7026 and NU7441. NU7026 is an ATP-competitive inhibitor of DNA-PKcs which displays selectivity over other PIKK family enzymes. It was developed using LY294002 as a template, shows a 60-fold greater potency against DNA-PKcs than PI3K, and is inactive against both ATM and ATR (IC50 0.23 μM for DNA-PK, 13 μM for PI3K, >100 μM for ATM and ATR). It has been shown to sensitize mouse embryonic fibroblasts and Chinese hamster ovary cells to radiation *in vitro*, and is not cytotoxic itself (68, 69). Additionally, NU7026 has shown chemosensitization activity, and has been shown to have synergistic cytotoxic activity when combined with chlorambucil in chronic lymphocytic leukemia cells, and this chemosensitization was found to correlate with inhibition of DNA-PKcs phosphorylation (70). In pancreatic ductal adenocarcinoma cells, inhibition of NHEJ with NU7026 has been shown to result in accumulation of DNA damage, inhibition of growth, and ultimately apoptosis even in the absence of exogenous DNA-damaging agents (71). Our previous data have used NU7026 to demonstrate the potential of DNA-PKcs inhibition as a chemosensitization strategy in HGSOC cell lines derived from patients with clinically platinum-resistant disease (37).

NU7441 is more potent than NU7026, and is a specific inhibitor of DNA-PKcs, with at least 100-fold selectivity for this enzyme compared to other PIKK family kinases (72, 73). NU7441 has been found to increase the response of many cancer cell types to both chemo- and radiotherapy (73–88). The specificity of this compound was initially illustrated by the finding that it increases chemo- and radiosensitivity in MO59 glioblastoma cells, but not in their DNA-PKcs-deficient MO59-J counterparts. However, the mechanism by which NU7441 increases drug sensitivity has recently been shown to also result from inhibition of multidrug-resistance protein 1 (MDR1), resulting in increased nuclear accumulation of cytotoxic agents that are substrates for this drug efflux pump and thus adding complexity to the mechanistic interpretation of response data obtained using this compound (85). Interestingly, MDR1 upregulation itself has been associated with DNA-PKcs activity in glioblastoma cell lines with acquired doxorubicin resistance (61).

To date, only one pharmacological inhibitor of DNA-PKcs has been assessed clinically. CC-115 is a dual DNA-PKcs/mTOR inhibitor which has been used in a phase 1 trial for patients with advanced malignancies (NCT01353625; http://clinicaltrials.gov). The results of this study are expected in 2016. DNA-PKcs has also been targeted in another recently completed phase 1 trial, combining DT-01, a DSB mimetic, with radiation in metastatic melanoma (NCT01469455). DT-01 is comprised of small DNA molecules, known as Dbait, which sequester DNA-PKcs and PARP, thereby impairing the DNA repair response to radiotherapy (89). Intriguingly, DNA-PKcs has recently been identified as key protein downregulated in breast cancer stem cells in response to treatment with the broad spectrum antibiotic doxycycline (90). Moreover, this was associated with increased radiosensitivity and decreased mammosphere formation *in vitro*, and suggests a potentially immediate way to incorporate DNA-PKcs inhibition into clinical practice.

## DNA-PKcs Inhibition as a Platinum Sensitization Strategy

Although the majority of HGSOC patients initially respond to platinum-based chemotherapy, the emergence of resistance is a major barrier to its long-term effectiveness. Therefore, identifying a targeted strategy to restore response may offer real promise in the treatment of this disease. As discussed here, DNA-PKcs inhibition has been shown to be effective in restoring platinum cytotoxicity in HGSOC (37). 4,5-Dimethoxy-2-nitrobenzaldehyde (DMNB), a cell-permeable vanillin derivative has also been reported to sensitize A2780 human ovarian carcinoma cells to cisplatin via inhibition of DNA-PKcs (91). Loss of DNA-PKcs has also been associated with increased cisplatin response in cervical cancer and gliomas (92, 93). The therapeutic activity of DNA-PKcs inhibition has also been linked to mechanisms independent of its role in DNA-damage repair, and with respect to restoring platinum sensitivity. This may partly be related to increased drug accumulation due to MDR1 protein inhibition; however, additionally, we have shown that DNA-PKcs is involved in DNA-damage-mediated activation of AKT cell survival signaling (37). In this study, we found that platinum exposure induces an AKT-dependent pro-survival DNA damage response in clinically platinum-resistant but not platinum-sensitive HGSOC. In this system, AKT is phosphorylated specifically on Ser473 by DNA-PKcs in the nucleus of platinum resistant but not sensitive cells. Inhibition of DNA-PKcs or AKT, but not mTORC2, was found to restore platinum sensitivity. Together, these data indicate that DNA-PKcs inhibition might be a clinical useful strategy for the prevention of platinum-induced AKT activation without interfering with normal glucose homeostasis. Although DNA-PKcs inhibitors are not yet ready for full clinical implementation, we have been able to test our laboratory hypothesis that preventing DNA-PKcs-mediated AKT signaling will reverse platinum resistance clinically by using an AKT inhibitor. A clinical trial led by our center reported a 37% overall response rate to daily oral AKT inhibition using GSK2110183 in combination with six cycles of 3-weekly carboplatin (AUC5) and paclitaxel (175 mg/m^2^) in clinically platinum-resistant ovarian cancer: a patient group with an expected response rate to carboplatin/taxol of only 10–14% (94).

*In vitro* studies have also shown the potential of DNA-PKcs inhibition as a sensitizing strategy for platinum compounds in other cancers, including osteosarcoma, breast, and pancreatic (37, 95, 96), and therefore development of such inhibitors may present broad clinical applicability. In particular, they may be particularly beneficial in lung cancer, where platinum resistance is also associated with aberrant DDR mechanisms (97). A role for DNA-PKcs in driving metastatic processes, i.e., angiogenesis, migration, and invasion, has been recently identified, suggesting an additional benefit of DNA-PKcs inhibitor-based chemotherapy in treating metastatic cancers (98).

The promise of PARP inhibition strategies and DNA-PKcs as a therapeutic target highlights the importance of understanding the clinical significance of the functioning and defective DNA repair mechanisms in cancer. There remains the need to identify reliable biomarkers of tumor cell response and resistance to therapies targeting DNA repair proteins and indeed to identify and validate new therapeutic targets from this critical but insufficiently mined resource. The identification of patient subgroups who will benefit most from such strategies is also required – DDR proteins such as DNA-PKcs have been suggested to have a tumor-suppressive role in the early stages of carcinogenesis where ineffective DDR may contribute to the generation of genomic instability that drives tumor progression (99). As such, the development of DNA-PKcs inhibitions, and indeed other DDR targeted therapies, should be mindful of DNA damage thresholds that can be either oncogenic or tumor-suppressive, depending on the tumor stage. Together, such knowledge and understanding will translate into the development of new directed strategies that will help overcome clinical platinum resistance in ovarian cancer, and by that reduce patient mortality.

## Conflict of Interest Statement

The authors declare that the research was conducted in the absence of any commercial or financial relationships that could be construed as a potential conflict of interest.
